# Hot Topics in the Surgical Treatment of Intrahepatic Cholangiocarcinoma: A Narrative Review of Current Managements

**DOI:** 10.3390/cancers17193127

**Published:** 2025-09-26

**Authors:** Silvio Caringi, Antonella Delvecchio, Annachiara Casella, Valentina Ferraro, Michele Dezio, Stefania Marini, Roberto Calbi, Francesco Cortese, Rosalinda Filippo, Matteo Stasi, Tommaso Maria Manzia, Michele Tedeschi, Riccardo Inchingolo, Riccardo Memeo

**Affiliations:** 1 Department of Surgery, Università Degli Studi Roma “Tor Vergata”, Via Montpellier 1, 00133 Rome, Italy; 2 Unit of Hepato-Biliary and Pancreatic Surgery, “F. Miulli” General Hospital, Acquaviva delle Fonti, 70021 Bari, Italy; a.delvecchio@miulli.it (A.D.); a.casella@miulli.it (A.C.); v.ferraro@miulli.it (V.F.); r.filippo@miulli.it (R.F.); matteo.stasi@miulli.it (M.S.); m.tedeschi@miulli.it (M.T.); r.memeo@miulli.it (R.M.); 3 Department of Radiology, “F. Miulli” General Hospital, Acquaviva delle Fonti, 70021 Bari, Italy; m.dezio@miulli.it (M.D.); s.marini@miulli.it (S.M.); r.calbi@miulli.it (R.C.); 4 Interventional Radiology Unit, “F. Miulli” General Hospital, Acquaviva delle Fonti, 70021 Bari, Italy; f.cortese@miulli.it (F.C.); r.inchingolo@miulli.it (R.I.); 5 Transplant and HPB Unit, Department of Surgery Sciences, University of Rome Tor Vergata, 00133 Rome, Italy; manzia@med.uniroma2.it; 6 Department of Medicine and Surgery, LUM University, Casamassima, 70010 Bari, Italy

**Keywords:** intrahepatic cholangiocarcinoma, liver tumor, liver neoplasm

## Abstract

Intrahepatic cholangiocarcinoma (iCCA) is the second most common primary liver cancer with a poor prognosis. Negative-margin resection is presently the only potentially curative treatment option. Emerging trends with direct applicability to surgical strategy include margin thickness, lymphadenectomy, optimization of future liver remnant (FLR), minimally invasive techniques, incorporation of systemic therapy, and reconsideration of liver transplantation. This review emphasizes areas of consensus and ongoing debate. Margins ≥5–10 mm are associated with improved results, but biology generally takes precedence over prognosis. Regional lymphadenectomy enhances staging accuracy, although its therapeutic benefit remains unsettled. PVE is standard for FLR enlargement, LVD provides faster hypertrophy, and ALPPS remains reserved for highly selective cases. Minimally invasive and robotic hepatectomy share oncologic results in skilled institutions. Systemic therapies, including immunotherapy and biomarker-directed targeted therapy, are increasingly being incorporated perioperatively. Liver transplant may be of potential value in early-stage disease or on strict indications after neoadjuvant treatment. The modern surgical management of iCCA encompasses a blend of oncologic considerations, FLR optimization, minimally invasive surgery, and systemic therapy according to tumor biology. Multidisciplinary planning and participation in clinical trials are necessary to align surgical innovation with advancements in molecular and systemic treatments, ultimately leading to improved long-term outcomes.

## Simple Summary

Intrahepatic cholangiocarcinoma (iCCA) is a rare liver cancer in the West, but its occurrence is increasing, and it has endemic incidence in Southeast Asia. Surgery remains the sole promise of cure, but resection is often overshadowed by liver function, tumor biology, and stage of disease. This review provides a comprehensive overview of the surgical management of iCCA from the surgeon’s perspective, covering fundamental issues such as optimal margins of resection, lymphadenectomy, minimally invasive resection, and strategies for maximizing the future liver remnant (PVE, LVD, ALPPS). It briefly discusses molecular profiling (e.g., IDH1, FGFR2) for selection of therapy, growing use of neoadjuvant and adjuvant therapy, and the changing conditional role of transplantation. Proper patient selection, multidisciplinary care, and the presence of high-volume institutions with advanced equipment are crucial. Personalized, biology-directed surgical methods are suggested to improve long-term results in the future.

## 1. Introduction

**Figure 1:**
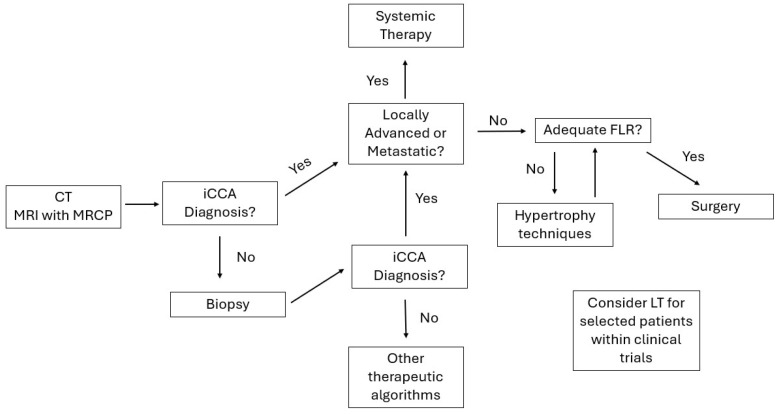
Flowchart of diagnostic and therapeutic pathways for iCCA.

Incident rates of iCCA have increased worldwide in recent decades, especially in Western countries, and iCCA remains hyperendemic in Southeast Asia, secondary to infection with liver fluke [1,2,3,4]. The outcomes are varied: median five-year survival after R0 resection ranges from ~25% to ~45% between series, depending mainly on stage at presentation, tumor biology, and adequacy of surgical clearance [5,6,7,8]. Surgical treatment is feasible in a limited number of patients at diagnosis, and the correlation between anatomical resectability, functional resectability, and preservation of proper FLR governs perioperative decision-making [9,10]. Figure 1 summarizes in a flowchart the diagnostic and therapeutic pathways for a patient with a liver lesion suspected of being an iCCA.

The purpose of this review is to provide a surgeon-focused, evidence-based overview of current issues in iCCA surgery. We highlight

1. The evidence supporting obligatory margin width and the effect of anatomical vs. non-anatomical resections on survival;
2. Staging and possible therapeutic indications of regional lymphadenectomy;
3. Present FLR optimization methods (PVE, LVD, ALPPS) and comparative outcomes;
4. Selection and method for minimally invasive and robotic hepatectomy;
5. Systemic therapy in adjuvant and neoadjuvant/conversion therapy, including immune checkpoint blockade and target therapies (IDH1, FGFR2);
6. The judiciously expanding role of liver transplantation;
7. Recurrence, monitoring, and treatment of recurrence.

Where possible, we quote consensus statements and randomized or high-quality comparative information. When data are retrospective or limited, we state the uncertainty and pragmatic safeguards [6,7,11,12,13,14].

## 2. Epidemiology, Risk Factors, and Natural History

A global rise in the rate of iCCA has been noted in Europe and North American population-based registries and attributed to augmented detection, classificatory changes, and changing risk factor prevalence like chronic viral hepatitis (HBV/HCV), metabolic dysfunction-associated steatotic liver disease (MASLD/NAFLD), diabetes, and obesity [1,2,3,4,15,16]. In Southeast Asian endemic regions, *Opisthorchis viverrini* and *Clonorchis sinensis* are the main carcinogens that coexist with nitrosamine exposure [2,3,17]. Autoimmune cholangiopathies, particularly primary sclerosing cholangitis (PSC), congenital biliary malformations (such as choledochal cysts and Caroli disease), and prior biliary-enteric anastomoses are established risk factors [18,19,20,21].

Long-term cholangiocyte injury induces a pro-oncogenic microenvironment characterized by oxidative stress and sustained NF-κB and STAT3 signaling activation [17]. Epigenetic deregulation and abnormal DNA methylation help in advancing driver mutation accrual. Next-generation sequencing has delineated into two broad molecular classes of iCCA: an “inflammation” type with excess cytokine signaling and an “proliferation” type with excess growth factor receptor activation [22]. Recurrently mutated genes are IDH1/2 mutations, FGFR2 fusions, and BAP1, ARID1A, and TP53 inactivation [23,24,25]. These lesions are of prognostic and therapeutic significance. For instance, FGFR2 fusions predict response to FGFR inhibition with agents such as futibatinib and IDH1 mutations predict susceptibility to IDH1 inhibition with ivosidenib [24,25]. Recognition of these molecular subgroups increasingly informs decisions for clinical trials, consideration for neoadjuvant targeted therapy, and risk stratification following resection.

Natural history is governed by hepatic reserve and tumor biology. In approximately one-third of patients, underlying cirrhosis exists, which restricts resection and elevates the risk of post-hepatectomy liver failure (PHLF) [26,27]. Multifocality, vascular invasion, satellite nodules, and nodal metastasis are among the strongest pathological predictors of unfavorable survival and premature recurrence [5,6,7,8,28].

## 3. Molecular Landscape and the Surgical Interface

Integrative genomic profiling has revealed that iCCA is molecularly heterogeneous, characterized by frequent alterations in IDH1/2, FGFR2, BAP1, ARID1A, TP53, and other genes, with distinct epigenetic and transcriptomic subtypes [22,29,30,31,32,33,34]. These are associated with clinicopathologic phenotypes (e.g., FGFR2 fusion-positive disease is commonly found in young non-cirrhotic individuals) and increasingly inform systemic therapy decisions [23,24,35,36]. Two groups of biomarkers have direct surgical relevance. First, IDH1-mutant iCCA (~15–20%) may possess distinct metabolic and epigenetic biology; ivosidenib improves progression-free survival in relapsed/refractory disease; and durable disease control on target therapy may render conversion surgery possible in carefully selected patients [23,37]. Second, FGFR2 rearrangements (~10–15%) are also FGFR inhibitor-sensitive (pemigatinib, infigratinib, futibatinib), occasionally leading to considerable tumor regression and surgical downstaging; however, resistance via gatekeeper mutations is common, and the durability of complete pathologic responses following targeted therapy remains to be systematically established [24,25,35,36].

From a surgical standpoint, molecular risk stratification allows for nuanced decision-making: the aggressiveness of resection and margin pursuit might be balanced against predicted systemic controllability; conversely, aggressive biology (i.e., TP53/KRAS mutations, high proliferative subtype) may require broader margins and more liberal nodal dissection where feasible or refer patients to trials and multimodal treatment [29,30,31,32,33].

## 4. Diagnosis, Staging, and the Role of Staging Laparoscopy

High-resolution multiphasic CT or MRI with MRCP is standard for local staging, supplemented by imaging of the chest to assess metastatic dissemination [9,10,25].

Despite the advancement in magnetic resonance cholangiopancreatography, ERCP remains a significant tool for diagnosing and preoperatively evaluating iCCA. ERCP facilitates intense cholangiographic imaging along with tissue procurement using brush cytology or forceps biopsy [12]. Although traditional cytology is not very sensitive, integration with fluorescence in situ hybridization or next-generation sequencing increases diagnostic yield significantly [38].

ERCP further facilitates therapeutic biliary tree drainage in those with cholangitis or obstructive jaundice, optimizing hepatic function before major hepatectomy [26]. Selective segmental drainage can be achieved in an attempt to preserve enough future remnant liver, particularly in perihilar disease. These benefits must be balanced against the risk of post-ERCP pancreatitis, cholangitis, and potential tumor seeding; prophylactic antibiotics and proper patient selection are indicated [12,38,39].

Endoscopic nasobiliary drainage (ENBD) constitutes a standby or adjuvant to internal stenting for preoperative biliary drainage. ENBD provides external continuous drainage and permits bile flow monitoring and repeated cholangiography or intraductal lavage without returning to endoscopic maneuvers [40]. In cholangitis or severe hyperbilirubinemia complicating iCCA, ENBD has been demonstrated to be related to lower post-drainage infection rates and is possible with a more controlled reduction in bilirubin levels before prolonged hepatectomy [41]. Although patient discomfort and risk of accidental dislodgement are undesirable factors, ENBD is particularly valuable when bilateral or multi-segmental drainage is required or in the case of early postoperative removal [40,41].

Single-operator digital cholangioscopy enables direct endoscopic visualization of biliary epithelium and directed biopsies. In suspected iCCA, particularly in the setting of indeterminate intrahepatic or hilar strictures on imaging, cholangioscopy increases diagnostic yield over standard brush cytology [42]. Endoscopic features of malignancy are irregular mucosa, friability, and abnormal vascular patterns, which can be biopsied under direct visualization [43].

Preoperative cholangioscopy holds two important surgical advantages. Firstly, it defines the longitudinal extent of intraductal spread of tumor, influencing the division level of the bile duct and the need for high-complexity biliary reconstruction. Secondly, it provides adequate tissue specimens suitable for histological and molecular assessment, facilitating inclusion in biomarker-driven clinical trials or consideration for neoadjuvant targeted therapy [42,43,44]. While its use for therapeutic purposes is limited to personalized palliative care interventions, its role as a preoperative map is more fully realized.

PET/CT is used irregularly and may be useful for detecting unknown nodal or extrahepatic disease. Serum CA 19-9 is nonspecific but can be useful in context for prognostic and response assessment [45]. A biopsy is not required before resection if imaging is normal and resectability is clear; however, a histological sample is necessary when neoadjuvant treatment will be administered or a differential diagnosis of hepatocellular carcinoma (HCC) is suspected [11,12].

While overall imaging findings and clinical presentation can provide background to primary resection without histological diagnosis in solitary, clearly resectable lesions, preoperative tissue diagnosis is recommended in a variety of settings. These include neoadjuvant or conversion therapy consideration, diagnostic dilemma between iCCA and hepatocellular carcinoma or metastatic adenocarcinoma, and participation in clinical trials requiring molecular characterization [7]. The tissue can be acquired with image-guided percutaneous core biopsy, ERCP-directed forceps biopsy, or cholangioscopy-guided sampling [12,38,43].

Histopathology not only diagnoses adenocarcinoma but also yields prognostically relevant information. Two variants, small-duct and large-duct, with distinct growth patterns, molecular and clinical behavior [46], have been identified. Immunohistochemical cytokeratin 7 and 19 staining supports biliary differentiation and iCCA from metastatic gastrointestinal adenocarcinoma differentiation [45]. More often than not, preoperative biopsy tissue undergoes molecular analysis for actionable mutations like IDH1/2 mutations or FGFR2 fusions, which directs targeted therapy and affects surgical planning if conversion of unresectable disease is to be achieved [22,24,25,35,47]. Histopathological and molecular information thus forms the basis of precision surgery as well as personalized systemic treatment.

Despite improvement, one-third or more of “resectable” patients have occult disease within the peritoneum or extrahepatic at the time of exploration; selective routine staging laparoscopy has been associated with avoidance of non-therapeutic laparotomy and improved perioperative parameters in current series [48,49,50]. Indications for routine risk factors necessitating staging laparoscopy are large tumors, high CA 19-9, and equivocal but suspicious peritoneal nodules [48,49].

## 5. Defining Resectability and Surgical Strategy

Resectability in iCCA is based on both oncologic clearance and preservation of adequate FLR. In non-cirrhotic livers, post-resection FLR ≥ 20–25% can be an acceptable criterion; in steatotic or chemotherapy-induced livers, ≥30–35%; and in cirrhosis (Child–Pugh A), ≥40% is typically sought [51,52,53]. Anatomic resection is preferable if it improves oncologic clearance along segmental inflow/outflow planes and facilitates en bloc lymphadenectomy; however, non-anatomic wedge resection is reasonable for small, peripheral tumors with favorable biology [6,7,54]. Vascular resection and reconstruction (portal vein or hepatic vein) can be indicated in the setting of locally advanced disease if it allows for R0 resection with an adequate FLR; rarely is biliary reconstruction required for peripheral iCCA, but it is an option for hilar-adjacent tumors [55,56,57]. Determinations should include estimation of PHLF risk (e.g., using liver function tests, indocyanine green clearance, or hepatobiliary scintigraphy) and institutional proficiency [51,52,53,58].

## 6. Margin Width: How Wide Is Wide Enough?

Margin status (R0 vs. R1) is among the most powerful surgically predictive determinants of survival in iCCA; however, the optimal margin size remains debatable. Various retrospective series and meta-analyses demonstrate improved OS and DFS with margins ≥ 5 mm, and further increase to ~10 mm if anatomically secure [5,6,7,8,59,60,61,62]. Incremental gain of more than ~10 mm is inconsistent, and tumor biology typically dominates the results, particularly in node-positive or multifocal disease [6,7,60].

Surgical advice is to try an R0 resection for ≥5–10 mm where feasible without jeopardizing FLR or vascular inflow/outflow. In situations where a wide margin compromises critical vasculature risk, parenchymal-sparing techniques in conjunction with adjuvant therapy may be an acceptable compromise [54,59,62]. A frozen section can guide intraoperative decision-making on bile duct margins, where relevant, though most iCCAs are parenchymal and do not require ductal transection [56,57].

Intraoperative ultrasound (IOUS) has thus become a critical instrument for liver surgery. High-frequency IOUS provides a real-time view of hepatic vasculature and parenchyma and can assist in identifying satellite lesions or tiny metastases not seen on preoperative imaging [13]. Several studies suggest that IOUS findings impact surgical planning in up to one-third of patients, typically by finding additional lesions or by better positioning the transection plane [14].

By correlating the tumor–vascular relationships within space, IOUS steers parenchymal transection to achieve adequate (>5–10 mm) margins with the preservation of an adequate future liver remnant [5]. It facilitates controlled vascular clamping and reduces the risk of important intraoperative hemorrhage. These advantages are cost-effectively translated to higher R0 resection rates and improved disease-free survival [2,22,63,64]. IOUS is now standard practice in high-volume hepatobiliary centers.

## 7. Regional Lymphadenectomy: Staging, Prognosis, and Potential Therapeutic Value

Nodal metastasis is an aggressive, poor prognostic factor in iCCA. Present practice increasingly supports routine regional lymphadenectomy (LND) for maximum staging, with a minimum harvest target of six nodes from the hepatoduodenal ligament and along the common hepatic artery [11,12,13,65,66]. Maximum nodal staging determines prognosis and the choice of adjuvant therapy (e.g., capecitabine) and may influence trial eligibility [67]. The survival benefit of LND remains unsettled. A few propensity-matched series show a therapeutic advantage—namely in hilum-proximal tumors with elevated nodal risk—while none show an OS advantage after stage and biology adjustment [65,68,69,70]. Nonetheless, LND is associated with low morbidity for skilled surgeons, increases the accuracy of staging, and is therefore reasonable as a default for the majority of resections, acknowledging that very vigorous dissections can increase lymphatic leak and biliary ischemia [65,69]. Sentinel mapping remains investigational [71].

## 8. Optimizing the Future Liver Remnant: PVE, LVD, and ALPPS

PHLF remains the leading cause of mortality after major hepatectomy. FLR growth avenues are central to modern iCCA surgery

Portal Vein Embolization (PVE): PVE triggers contralateral lobe overgrowth within 3–6 weeks and is the historical gold standard; it is a safe, universally available, and evidence-based procedure based on long-term experience [72,73,74].

Liver Venous Deprivation (LVD): The combination of portal and hepatic vein embolization (frequently right portal + right/segmental hepatic veins) accelerates and increases hypertrophy compared to PVE, increasing resection rates with no deficit in safety in comparative trials and meta-analyses [63,64,75,76]. LVD is attractive when shortening surgery time or where PVE-hypertrophy will be insufficient.

Associating liver partition and portal vein ligation for staged hepatectomy (ALPPS): ALPPS yields rapid hypertrophy and excellent resection margins but at the cost of higher morbidity and classically higher mortality, especially with initial experience; correct selection, technical refinement, and ERAS protocols have minimized outcomes, but ALPPS will need to stay reserved for very selected patients in high-volume centers [77,78,79,80].

The choice among PVE, LVD, and ALPPS is determined by the baseline FLR, urgency, tumor biology (risk of progression), the center’s expertise, and the patient’s comorbidities. Quantitative liver function tests (e.g., 99mTc-mebrofenin scintigraphy) and volumetry guide thresholds and enable personalized choices [51,58,75].

## 9. Minimally Invasive and Robotic Hepatectomy

Minimally invasive liver resection (MILR)—robotic and laparoscopic—has come a long way. In carefully selected iCCA, MILR is associated with less blood loss, reduced analgesia requirements, and shorter postoperative hospitalization, with equivalent R0 rates and perioperative morbidity in high-volume institutions [81,82,83,84,85]. Robotic platforms offer improved dexterity during hilar dissection and LND, but they possess steep learning curves, and achieving oncologic equivalence requires adherence to anatomical principles and nodal clearance goals [83,86].

Key caveats include proper selection of the patient (tumor size, relationship to major vasculature), maintenance of oncologic technique (no compromise in margin or LND), and readiness to convert without unnecessary delay. The absence of tactile feedback is offset by high-definition imaging and accurate ultrasound application [81,82].

## 10. Complex Resections: Vascular and Biliary Reconstruction

Well-selected patients with locally advanced disease also benefited from vascular resection and reconstruction to achieve R0 margins. Resection of the portal vein with primary anastomosis or interposition graft, and hepatic vein reconstruction in cases of segmental outflow obstruction, can be achieved with acceptable morbidity in experienced centers [55,56,57,87]. Biliary reconstruction is less common than in perihilar cholangiocarcinoma but is rarely needed for lesions invading the second-order ducts; Roux-en-Y hepaticojejunostomy is the standard technique [56]. Complex resections should be relegated to high-volume hepatobiliary centers because of volume–outcome relationships for extensive hepatectomy and complex reconstructions [88,89].

## 11. Perioperative Care, ERAS, and Prevention of PHLF

Improving Recovery After Surgery (ERAS) pathways tailored to hepatectomy reduce complications and hospital stay, with an emphasis on multimodal analgesia, judicious fluid administration, early mobilization, and nutrition [90,91]. Prevention of PHLF involves careful attention to prehabilitation (including nutrition and sarcopenia), management of cholestasis and cholangitis, and prevention of small-for-size syndrome. Intraoperative strategies include low central venous pressure anesthesia, selective inflow control, parenchymal transection technologies, and meticulous hemostasis [90,91,92,93]. Postoperatively, early recognition of liver dysfunction using dynamic tests and algorithms directs timely intervention [51,58].

## 12. The Role of Chemotherapy

Systemic therapy is one of the cornerstones in the treatment algorithm for intrahepatic cholangiocarcinoma (iCCA), particularly for those with unresectable or metastatic disease, or recurrence after curative-intent resection. Over the last decade, we have witnessed significant advances that have transformed the treatment landscape, integrating cytotoxic therapy with molecularly targeted and immunotherapeutic strategies.

With high recurrence risk (>50% at two to three years), adjuvant treatment is critical. The randomized BILCAP trial set capecitabine as an established adjuvant treatment for resected biliary tract cancer, with evidence of OS benefit on sensitivity/per-protocol analyses and durable benefit on long-term follow-up despite intention-to-treat result complexities [94,95,96,97]. Real-world data suggest a greater benefit in high-risk characteristics (R1, N+, large tumor), although heterogeneity remains [98,99]. Several trials are exploring adjuvant immunotherapy or targeted therapy; their outcomes are awaited. Meanwhile, six months of capecitabine is usually favored in fit patients, particularly with nodal disease or close/four-margin disease [11,12,13,95,96,97].

The gemcitabine and cisplatin combination has been established as the first-line standard after the seminal phase III ABC-02 trial, which revealed a median overall survival (OS) of 11.7 months compared with 8.1 months for gemcitabine alone [100]. The doublet remains the backbone standard in Western practice and has been reaffirmed in real-world cohorts across different geographic regions [100]. Gemcitabine–oxaliplatin (GEMOX) is an alternative in cisplatin-intolerant or contraindicated patients, such as renal insufficiency, with comparable response rates and a better administration schedule [101]. Nab-paclitaxel plus gemcitabine–cisplatin has shown significant activity in phase II trials, with >40% objective response rates and approximately 19-month median OS, although confirmation in the phase III setting is pending [102].

In borderline-resectable or initially unresectable iCCA, neoadjuvant systemic treatment may select the biology and achieve downstaging. In metastatic biliary cancer, the addition of durvalumab to gemcitabine/cisplatin (TOPAZ-1) improved OS and rapidly became a first-line backbone; this combination is frequently employed off-trial in iCCA conversion strategies with encouraging disease control rates [100,101,103,104]. In biomarker-selected patients, IDH1 inhibitors and FGFR inhibitors have the capability to produce radiographic responses, potentially allowing for subsequent resection; the duration of these responses and pathologic correlation with surgery require further investigation [24,35,36,37,102].

Until recently, second-line therapy has had a limited evidence base. The ABC-06 trial demonstrated a survival benefit with modified FOLFOX (oxaliplatin, leucovorin, and fluorouracil) compared with best supportive care (median OS 6.2 vs. 5.3 months), establishing FOLFOX as the standard second-line therapy in fit patients [105]. In patients who are intolerant to oxaliplatin, fluoropyrimidine-based regimens like capecitabine or 5-fluorouracil with leucovorin remain appropriate alternatives, with reduced rates of response [106].

Genomic profiling has also revealed actionable alterations in a subset of iCCA patients, allowing for the integration of targeted drugs into chemotherapy regimens. FGFR2 fusions and rearrangements, which are found in approximately 10–15% of iCCA, are treatable with selective FGFR inhibitors such as pemigatinib and futibatinib, both of which achieved 35–42% objective response rates in phase II trials [25,35]. Similarly, mutations in IDH1 may be targeted by ivosidenib, which prolonged progression-free survival compared to placebo in the phase III ClarIDHy trial [23]. Although these medications are typically employed after first-line chemotherapy, clinical trials are investigating their administration with gemcitabine–cisplatin up front.

Immune checkpoint inhibitors are also transforming the therapeutic landscape. The phase III TOPAZ-1 trial proved that the addition of durvalumab, a PD-L1 inhibitor, to gemcitabine–cisplatin clearly improved OS (median 12.8 vs. 11.5 months) without uncontrolled toxicity, and chemo-immunotherapy became a new standard of care [99]. Early-phase combination trials of checkpoint inhibitors with targeted agents or other cytotoxics are underway and will likely continue to personalize treatment algorithms.

## 13. Locoregional Therapies

Locoregional treatments have become important tools in the multidisciplinary approach to intrahepatic cholangiocarcinoma (iCCA) in patients with unresectable disease who are not candidates for transplantation and in patients with limited intrahepatic recurrence. These modalities are designed to achieve local tumor control, enhance survival, and, in the appropriate patient, downstage disease to permit curative resection or transplantation.

Transarterial chemoembolization (TACE) delivers high concentrations of chemotherapeutic agents directly into the hepatic artery of the tumor supply, with subsequent embolic particles inducing ischemic necrosis. Although TACE is an established modality for the treatment of hepatocellular carcinoma, its role in the management of iCCA is uncertain. Small prospective trials and retrospective cohorts have reported median total survival of 12–22 months in unresectable iCCA, with a response rate of 20–40% [107,108,109]. Drug-eluting bead TACE delivers more controlled drug release and has been demonstrated to be as effective with reduced systemic toxicity compared to standard TACE [107]. Careful patient selection, typically with preserved liver function and limited extrahepatic disease, is crucial to achieve benefit and avoid complications like post-embolization syndrome or hepatic insufficiency [108].

Transarterial radioembolization (TARE) with yttrium-90 (Y-90) microspheres has increasing applications in the management of unresectable iCCA. TARE delivers high doses of intra-arterial radiation with sparing of the surrounding parenchyma. Prospective and retrospective series have a median survival of 12–22 months and >80% disease control rates [110,111,112]. In some series, TARE has been utilized as a bridge to surgery or transplant by a reduction in tumor burden [111]. Its safety is excellent with low post-embolization syndrome rates compared to TACE. Present randomized trials are comparing TARE with systemic chemotherapy to define its role as a first-line or combination therapy.

Radiofrequency ablation (RFA) and microwave ablation (MWA) are established treatments of small hepatocellular carcinomas and are increasingly applied in iCCA lesions ≤ 3 cm. RFA induces coagulative necrosis due to heat energy, whereas MWA produces more uniform and larger ablation sizes with shorter procedure times [113]. In isolated iCCA nodules in non-surgical candidates, local control rates of 60–80% and median survival of 20–30 months are achieved [113,114]. MWA appears to achieve higher levels of complete ablation and fewer local recurrences than RFA in some comparative studies [114]. Lesion size, proximity to large vessels, and liver function behind the lesion remain predictors of success.

Advances in the delivery of radiation, such as stereotactic body radiotherapy (SBRT), allow for the delivery of high biologically effective doses with very minimal exposure to surrounding liver tissue. SBRT has achieved 70–90% local control with 12–22 months median overall survival in unresectable iCCA [115,116]. SBRT is also employed as a bridge to resection or as a combined modality treatment with systemic chemotherapy. Uninvolved liver and organs at risk dose limitations are the most significant factors to minimize radiation-induced liver disease and gastrointestinal toxicity [115].

Hepatic arterial infusion chemotherapy (HAIC) is the delivery of high doses of cytotoxic drugs directly into the liver via an implanted pump or catheter. Phase II trials have yielded objective response rates of 20–40% and a median survival of 20 months in iCCA patients with refractory systemic therapy [117,118]. HAIC is most attractive in patients with liver-predominant disease and minimal extrahepatic spread, and ongoing trials are evaluating its use with systemic chemotherapy or immunotherapy.

## 14. Liver Transplantation: Conditional Options

Liver transplant (LT) for iCCA was earlier contraindicated due to high recurrence. Two controlled settings have recurred: (1) very-early iCCA (single lesion ≤ 2 cm) in cirrhotic livers, where 5-year survival comparable to HCC has been attained under strict selection; (2) unresectable yet liver-limited iCCA following aggressive neoadjuvant regimens with evidence of long-term disease control [119,120,121,122,123,124,125,126]. A multicenter series reported 50–70% 5-year OS in extremely selected patients with nodal/extrahepatic disease exclusion and favorable biology [122,123,124,125,126].

These results remain protocol-dependent and center-dependent; outside trials or predetermined criteria, LT for iCCA can only be considered experimental. Extensive staging (staging laparoscopy) and biomarker-guided risk assessment are crucial in planning [121,122,123,124]. All ongoing trials on LT for iCCA are summarized in Table 1.

## 15. Patterns of Recurrence, Treatment of Relapse, and Surveillance

Recurrence after resection is common and often intrahepatic, typically within the initial 12–18 months. Early recurrence indicates particularly poor outcomes and most likely reflects evidence of occult micrometastatic disease during surgery [5,6,7,8,28,127]. Management of relapse depends on distribution and biology. For liver-alone or oligometastatic recurrence, reoperation, ablation, or SBRT can be of substantial disease control in selected patients, whereas systemic treatment is first-line for widespread relapse [109,128,129,130,131].

Surveillance is typically performed using cross-sectional liver scans and chest imaging every 3–4 months for the first two years and then every 6 months to 5 years and CA 19-9 as an ancillary marker [11,12,13,45]. Risk-adjusted strategies by stage of presentation, node, margin, and molecular features are under investigation, including liquid biopsy approaches for the detection of minimal residual disease [132,133,134].

## 16. Special Scenarios and Populations

1. Cirrhosis and portal hypertension.

Portal pressure measurement in cirrhotic patients (HVPG, varices, thrombocytopenia) and dynamic liver function testing directs safe resection margins; parenchymal-sparing techniques and robust FLR growth pathways are required [51,52,53].

2. Elderly and frail.

Biological, rather than chronological, age, sarcopenia, and comorbidities influence outcomes; prehabilitation and ERAS maximize resilience [90,91,92,93].

3. Cholangitis and biliary sepsis.

Preoperative biliary drainage may be indicated for cholestasis with segmental duct–adjacent tumors, balanced against the risk of infection [56,57].

4. Secondary hepatectomy.

Recurrence intrahepatic only is curative in the minority with secondary resection; MILR can permit adhesiolysis-preserved reoperations in experienced hands [81,82,83,84,85,131].

## 17. Health Systems, Volume–Outcome Relationships, and Equity

High-volume centers are associated with lower perioperative mortality and improved long-term outcomes after major hepatectomy and complex biliary surgery [88,89]. Multidisciplinary tumor boards, including hepatobiliary surgery, interventional radiology, hepatology, pathology, medical oncology, and radiation oncology, are necessary for optimal staging and candidacy decisions. Access to biomarker testing (NGS), clinical trials, and advanced FLR optimization (LVD, ALPPS) remains unequal across regions, with equity disparities directly impacting resectability and survival [63,64,75,76,77,78,79,80,88,89].

## 18. Future Directions

Priorities for future research include the following: (1) prospective studies defining margin width thresholds by molecular subtype and nodal status; (2) randomized trials of LVD vs. PVE in iCCA with standardized functional outcomes; (3) stricter selection criteria and perioperative protocols for ALPPS; (4) neoadjuvant immunotherapy/targeted combination trials with pathologic response endpoints; (5) inclusion of circulating tumor DNA to guide adjuvant therapy and surveillance; and (6) harmonized definitions and quality measures for LND adequacy in minimally invasive resections. Coordination between surgical, medical, and translational teams will be crucial to move beyond anatomy-only models to biology-guided surgery.

## 19. Conclusions

Healing in iCCA remains focused on surgery but now necessitates far greater sophistication than merely technical resection. The modern surgeon must master nodal staging and margin science, apply FLR optimization wisely, derive an advantage from minimally invasive platforms with oncologic integrity, and integrate systemic and locoregional therapy tailored to molecular profiles. Through disciplined patient selection, methodical high-quality lymphadenectomy, and proactive use of PVE/LVD (reserving ALPPS for outlier cases), perioperative outcomes can be maximized. Likewise, multidisciplinary planning and clinical trials must be employed to translate emergent biologic concepts into durable improvements in survival. The emergent conditional role for liver transplant and biomarker-guided conversion strategies indicates what is possible when anatomy and biology are harmonized.

## Figures and Tables

**Table 1:** Ongoing trials of LT in iCCA.

TRIAL	TRIAL ID	STUDY	INCLUSION CRITERIA	PRE-TRANSPLANT	TYPE OF	PRIMARY	STATUS
**LIRICA**	NCT06098547	Prospective, Exploratory, Single-arm	Unresectable, histologically confirmed iCCA; Stable or partial response after ≥6 months of chemotherapy; No extrahepatic or nodal disease	Gemcitabine + Cisplatin ≥ 6 months; PET-MRI; Staging laparotomy with lymphadenectomy	Deceased or living donor	3-year and 5-year OS; DFS; Recurrence; Morbidity; QoL.	Ongoing Estimated end: 2028
**LIVINCA**	NCT06539377	Prospective, Non-randomized	Unresectable iCCA, stable or regressed after chemotherapy and/or locoregional therapy	GemCIS or FOLFOX; SIRT (Y-90 radioembolization) or ablation	Living donor only	Safety; DFS; OS; Dropout rate.	Ongoing Estimated end: 2030
**TESLA**	NCT04556214	Exploratory, Monocentric, Open-label	Unresectable iCCA, no vascular invasion, no extrahepatic or nodal metastases; Disease stability ≥ 6 months on systemic or locoregional therapy	GemCis or FOLFOX and/or SIRT or local ablation	Non-specified	OS from screening; OS from recurrence; DFS.	Ongoing Estimated end: 2030
**TORONTO (Early iCCA)**	NCT02878473	Prospective, Single-arm	iCCA ≤ 2 cm in cirrhotic patients; No extrahepatic spread; Negative lymph nodes	Active surveillance or selective downstaging	Deceased or living donor	5-year OS and cumulative recurrence risk.	Ongoing Estimated end: 2029
**TORONTO (Advanced/** **Stable iCCA)**	NCT04195503	Prospective, Single-arm, Open-label	Advanced unresectable iCCA; Disease stability ≥ 6 months under chemotherapy; No nodal or extrahepatic metastases	Gemcis or alternative regimens; Imaging every 3 months	Living donor only	1-year and 5-year DFS and OS.	Ongoing Estimated end: 2031
**iCOLA**	NCT06862934	Multicentric	Unresectable iCCA with stable disease or response after downstaging with systemic and locoregional therapy	Chemotherapy + Radioembolization (Y-90); Patient selection post-treatment	Non-specified	2-year and 5-year DFS and OS; LT conversion rate	Ongoing Estimated end: 2027

## Data Availability

No data were created.
